# A Descriptive Mixed-Methods Analysis of Sexual Behavior and Knowledge in Very Young Children Assessed for Sexual Abuse: The ASAC Study

**DOI:** 10.3389/fpsyg.2018.02716

**Published:** 2019-01-09

**Authors:** T. F. Vrolijk-Bosschaart, S. N. Brilleslijper-Kater, E. Verlinden, G. A. M. Widdershoven, A. H. Teeuw, Y. Voskes, E. M. van Duin, A. P. Verhoeff, M. de Leeuw, M. J. Roskam, M. A. Benninga, R. J. L. Lindauer

**Affiliations:** ^1^Department of Social Pediatrics, Child Abuse and Neglect Team, Emma Children’s Hospital, Academic Medical Center, Amsterdam, Netherlands; ^2^Department of Epidemiology, Health Promotion and Healthcare Innovation, Public Health Service, Amsterdam, Netherlands; ^3^Department of Medical Humanities, VU University Medical Center, Amsterdam, Netherlands; ^4^Department of Child and Adolescent Psychiatry, Academic Medical Center, Amsterdam, Netherlands; ^5^Former Medical Student, University of Amsterdam, Amsterdam, Netherlands; ^6^Department of Pediatric Gastroenterology, Emma Children’s Hospital, Academic Medical Center, Amsterdam, Netherlands; ^7^De Bascule, Academic Center for Child and Adolescent Psychiatry, Amsterdam, Netherlands

**Keywords:** sexual behavior, sexual knowledge, sexual abuse, children, evaluation, interviewing

## Abstract

Child sexual abuse (CSA) is a worldwide problem with serious consequences. We hypothesized that worrisome sexual behavior and knowledge would frequently be reported in children assessed after CSA. We therefore investigated (A) what types of sexual behaviors and knowledge were reported by parents of young children assessed for CSA; (B) in what cases such behaviors and knowledge were worrisome; and (C) how such children responded verbally and non-verbally during child interviews. We conducted a mixed-methods study, including qualitative inductive content analysis and quantitative analysis. It included 125 children (76 boys, 60.8%; median age 3.3 years, age range 0–11), all involved in the Amsterdam sexual abuse case (ASAC) and examined for highly suspected (*n* = 71) or confirmed CSA (*n* = 54). We identified themes from (1) the parent reports: sexual behavior (e.g., self-stimulation, touching others, imitation of sexual acts), fears and anxiety with regard to sexuality, and sexual utterances (sexual slang, references to sexual acts); and (2) the child interviews: behavioral reactions (avoidance, distractive behaviors), emotional reactions (anger, aggression), and verbal reactions (conspicuous utterances, refusal to talk about specific subjects). In 37% of the children the sexual behavior was deemed worrisome or very worrisome. Clinicians who assess children for CSA are advised to focus in particular on sexual behavior problems and inappropriate sexual knowledge.

## Introduction

Child sexual abuse is a common phenomenon all over the world. A meta-analysis has found high heterogeneity in the results of studies assessing CSA prevalence worldwide, ranging from 8 to 31% for girls and 3 to 17% for boys (Barth et al., 2013). Overall, girls appear more likely to become victims of CSA (Stoltenborgh et al., 2011; Barth et al., 2013).

Traumatic experiences in childhood, including CSA, may have short-term and long-term consequences. Short-term effects may be physical (such as injuries, infections, anogenital symptoms) or psychosocial (learning and concentration problems, internalizing, externalizing, sexual behavior problems) (Kendall-Tackett et al., 1993; Thompson et al., 2014; Lewis et al., 2016). In the longer term, CSA is associated with psychiatric disorders (including depression, eating disorders, and post-traumatic stress disorder), suicide attempts, self-destructive behaviors, and functional and somatic syndromes (Browne and Finkelhor, 1986; Beitchman and Zucker, 1991; Ehrensaft, 1992; Bremner, 2003; Dube et al., 2005; Maniglio, 2009, 2010, 2013; Chen et al., 2010; Afari et al., 2014).

A meta-analysis of 13 studies (including studies on preschool children) reported that 28% of sexually abused children have sexual behavior problems (Kendall-Tackett et al., 1993). Conversely, high percentages of children with sexual behavior problems have been found to have histories of sexual abuse (Chaffin et al., 2008; Allen, 2017) and therefore it is important to evaluate sexual behavior in children in whom CSA is suspected, merely to investigate whether there is an indication for treatment. Sexual behaviors are considered to be sexual problems if they appear more frequently or at a much earlier age than would be developmentally expected, are intrusive, occur with coercion, intimidation, or force, or are associated with emotional distress (Silovsky and Niec, 2002; Chaffin et al., 2008; Kellogg et al., 2009). However, there is no one specific sexual behavior that is indicative of sexual abuse (Kellogg et al., 2009), and other origins of such behaviors, including physical abuse, family violence, and other types of maltreatment, are known to be possible (Brilleslijper-Kater et al., 2004; Chaffin et al., 2008; Kellogg et al., 2009; Everson and Faller, 2012; Allen, 2017).

Over the past few decades, considerable attention has been focused on children’s sexual behaviors in relation to abuse. Many studies involved older children (Kellogg and Menard, 2003; Allen, 2017), younger children referred with sexual behavior problems (Silovsky and Niec, 2002; Baker et al., 2008), normative populations (Friedrich et al., 1998), or preselected CSA victims (Vrolijk-Bosschaart et al., 2018), or were review studies that can be considered outdated (Kendall-Tackett et al., 1993). Studies among children with suspected CSA, for instance in clinical samples, are rare.

Assessment of children’s sexual knowledge in child interviews has likewise received insufficient attention (Brilleslijper-Kater et al., 2004). A child interview, preferably performed by a child behavioral specialist or someone trained in the forensic interviewing of children, provides opportunities for the child to disclose abuse and for the researcher to assess a child’s sexual knowledge and verbal and non-verbal responses (Brilleslijper-Kater and Baartman, 2000; Brilleslijper-Kater et al., 2004). In the evaluation of suspected CSA, a child’s sexual knowledge is possibly an aspect equally important to analyze as sexual behavior (Brilleslijper-Kater and Baartman, 2000; Brilleslijper-Kater et al., 2004). Studies assessing sexual behavior and knowledge in preschool children with suspected CSA, and male children in particular, are limited (Lindauer et al., 2014).

In 2010, in response to a digital child pornography investigation in the United States, an Amsterdam daycare employee was suspected of having sexually abused dozens of young children. Many very young children, most of them boys, were considered possible victims. Police decrypted child pornographic images, and the employee eventually confessed to the sexual abuse of 87 children. During interrogation, detailed descriptions about the time span, frequency and type of abuse for each child were given by the employee. After parents of 20 children decided against pressing charges, the daycare worker was convicted of abusing 67 children. The ASAC is the largest confirmed CSA case in history involving one serial perpetrator. The ASAC is a unique case, owing to its large scale, the predominance of very young children, the predominance of boys, the confessed and convicted perpetrator, the high level of evidence, and the detailed documentation available about the abuse (Lindauer et al., 2014). Two previous papers have been published on the same cohort of children (Vrolijk-Bosschaart et al., 2017a,b). The previous papers focus on reported physical symptoms and psychosocial problems and the outcomes of the physical examination (Vrolijk-Bosschaart et al., 2017a,b). All together they report on the complete (multidisciplinary) pediatric assessment which was done shortly after the ASAC came to light.

We hypothesized that sexual behavior problems and age-inappropriate sexual knowledge would be frequently reported for the children involved in the ASAC. We therefore carried out a descriptive, mixed-methods study to investigate (A) what types of sexual behaviors and knowledge were reported by parents of young children assessed for CSA; (B) in what cases such behaviors and knowledge were worrisome; and (C) how young children assessed for CSA responded verbally and non-verbally during child interviews.

## Materials and Methods

### Study Setting

After the ASAC came to light, 130 young children for whom there was a suspicion of CSA received a pediatric evaluation by an urgently assembled multidisciplinary team at an emergency outpatient department (henceforth OPD) in the Emma Children’s Hospital of the Academic Medical Center (AMC) in Amsterdam. All parents of children referred to the emergency OPD were asked for informed consent to participate in this study.

As the perpetrator was still being questioned by the police, it was not yet certain which children had definitely experienced CSA. It took months for police to identify the children with confirmed CSA.

The pediatric evaluations were carried out by one of five multidisciplinary teams, each composed of a pediatrician, a social worker, and a child behavior specialist. A pediatric evaluation consisted of a full medical history in consultation with parents (including the child’s sexual behavior and knowledge), an anogenital examination, screening for sexually transmitted infections and pregnancies (if applicable), and interviews with the child (Jenny and Crawford-Jakubiak, 2013; Adams et al., 2016). The reported physical symptoms and psychosocial problems and the outcomes of the physical examination have been discussed in our previous papers (Vrolijk-Bosschaart et al., 2017a,b). The medical history, taken by the pediatrician in consultation with parents, was semi-structured (combining the standard medical interview with an interview topic guide). One focus in the topic guide was on sexual behavior and knowledge exhibited by the child and observed by the parents.

Children were additionally assessed in a private child interview, contingent on parental consent and the child’s age and language development (generally for children older than 2½ to 3 years), employing the Sexual Knowledge Picture Instrument (SKPI). The SKPI is a developmentally sensitive tool that tests young children’s sexual knowledge and assesses non-verbal signs of CSA (Brilleslijper-Kater and Baartman, 2000; Brilleslijper-Kater, 2005). The instrument consists of 14 child-friendly drawings to assess sexual knowledge. The children were interviewed using a semi-structured interview protocol containing exclusively open, non-leading questions. For each drawing, the children were first asked an open-ended question (such as “What does this picture show?”) followed by more structured questions if necessary (“What are the people doing?”). The people depicted are intended to represent familiar home situations for the children, with adults looking 25 to 40 years of age and the children resembling preschoolers. This child-friendly format enables assessment of the child’s knowledge of genital differences, gender, body parts and functions, adult sexual behavior, and differences between physical intimacy and sexual interactions. The SKPI has proven a useful instrument for children with some verbal abilities and is therefore suitable for children aged 3 or older who have a normal level of speech development (Brilleslijper-Kater, 2005).

### Study Design

We performed a retrospective mixed-methods study in a clinical sample of children assessed for suspected CSA, approved by the Medical Ethics Committee of the AMC hospital. We divided this research in three sections A, B, and C), to address our research questions:

(A) we performed a qualitative analysis of sexual behaviors and knowledge reported by parents during the taking of the full medical history (the medical interview);
(B) we quantitatively assessed the frequency of parent-reported non-worrisome and worrisome sexual behaviors and knowledge as recorded in the OPD medical files;
(C) we performed a qualitative analysis of the verbal and non-verbal responses in the child interviews.

In the following paragraphs we will refer to the above sections. During this analysis, the researchers were blinded to the police information (as the clinicians had been during the actual assessments).

### Data Extraction (Section A and C)

For the qualitative analysis (sections A and C), we accessed the children’s medical files to extract data for the inductive content analysis (Elo and Kyngäs, 2008). The analysis was performed by two independent investigators (TFVB, SNBK), who were blinded to the police information. The files contained parents’ reports on their child’s medical history, including the child’s sexual behaviors and sexual knowledge (henceforth “parent reports”), as well as clinical information based on the child interviews (“child interviews”).

Another investigator (EV) collected information about the nature and frequency of the abuse from the police reports; this was not present in the children’s medical files.

### Data Analysis (Section A and C)

#### Qualitative Analysis of the Medical Files (Section A and C)

The qualitative analyses were performed both manually and with MAXQDA, version 11. During the analyses the investigators were blinded to the police information. The contents of the files were analyzed in 3 steps, using inductive content analysis (Elo and Kyngäs, 2008):

##### Independent open labeling of file data

Two investigators with different professional perspectives: TFVB, a physician (medical perspective), and SNBK, a child behavior expert specialized in child abuse and neglect (psychosocial perspective) labeled the file data independently. This enabled analytic negotiation to gain a richer understanding of the data and reduce bias in the final analysis. Any disagreements about coding were resolved by consensus with other co-authors.

##### Grouping of labels (TFVB and SNBK in consultation)

Descriptions that appeared to pertain to the same phenomena were grouped together; all groupings were derived from the file data. Groupings were allowed to emerge organically. At this point in the analysis, we intentionally did not analyze results within an existing theoretical framework.

##### Condensation of data (TFVB and SNBK in consultation)

Groupings containing similar events or incidents were combined into categories, and categories were combined into themes. Themes were again allowed to emerge organically. Prominent themes were organized into hierarchies, and themes that emerged consistently across the sample were given emphasis. Subsequently the themes were presented for discussion to some co-authors (RJLL, YV, and GAMW). Sexual behaviors and knowledge pertaining to more than one theme were presented for discussion and categorized in the most appropriate theme. Relevant quotes (literal excerpts) from children’s medical files were selected to illustrate the themes. After the selection, the quotes were linked to the police information on whether a child was a confirmed or suspected victim.

### Establishing Criteria to Differentiate Between Non-worrisome and Worrisome Sexual Behaviors and Knowledge (Section B)

To address the quantitative research question (section B), we needed clear criteria to differentiate between non-worrisome (typical or age-appropriate) and worrisome (atypical or age-inappropriate) sexual behaviors and knowledge deriving from the parent reports. To establish these criteria we consulted five experts: two child behavior specialists (SNBK, EV), two pediatricians (AHT, LB), and one child psychiatrist (RJL). All five experts, all blind to police information, independently assessed summaries of the medical files of all children involved in the ASAC [a process described in more detail in our previous paper (Vrolijk-Bosschaart et al., 2017b)]. In two FGDs designed to determine whether the experts could distinguish confirmed victims from suspected ones, they discussed what indications were considered more worrisome for CSA and why. To enhance the reliability of our criteria, the expert opinions were then integrated with the characteristics of sexual behavior problems as described by Kellogg and the Committee on Child Abuse Neglect of the American Academy of Pediatrics in 2009 (Kellogg et al., 2009). This resulted in the criteria in Table 1. These criteria were used for the quantitative analysis of non-worrisome and worrisome sexual behavior and knowledge (B).

**Table 1:** Criteria for rating child sexual behaviors reported by parents.

Non-worrisome behavior ^1^	Somewhat worrisome behavior ^2^	Worrisome behavior ^3^	Very worrisome behavior ^4^
Occasional touching of own genitals, not autoerotic in nature	Rubbing body against others	Asking peer or adult to engage in specific sexual act(s)	Any sexual behaviors that involve children 3 or more years apart or at different developmental levels
Viewing or touching genitals, confined to close family members or peers	Trying to insert tongue in mouth while kissing	Inserting objects into genitals	Variety of sexual behaviors displayed on a daily basis
Showing own genitals, confined to close family members or peers	Touching peer or adult genitals or breasts	Explicitly imitating sexual acts (e.g., intercourse, oral copulation)	Sexual behavior that results in emotional distress or physical pain for the child and/or others
Standing or sitting too close	Sexually suggestive posing	Expression of anxiety related to sexuality	Expression of other physically aggressive behaviors related to sexuality
Trying to view peer or adult nudity	Prudish behavior	Clearly deviant defecation-related problems, such as poop smearing	Sexual behaviors that involve coercion
Behaviors are transient, infrequent, distractible, and confined to close family members or peers	Mimicking of movements that could be associated with sexual acts	Touching animal genitals	Display of age-inappropriate sexual knowledge
	Sexual behaviors that are occasionally but persistently disruptive to others, but transient and moderately responsive to distraction	Sexual behaviors frequently disruptive to others, persistent or obsessive, resistant to parental distraction, or autoerotic in nature	Behaviors are persistent and child becomes angry or upset if distracted.

*^*1*^Symptoms raise no concerns, and especially not for CSA. No further steps would be considered necessary to assess child for CSA; ^*2*^Symptoms do not raise concerns for CSA. Assessment is recommended of situational factors (family nudity, child care, new sibling, etc.) contributing to behavior, as well as the monitoring of symptoms over time; ^*3*^Symptoms raise concerns for CSA, though other diagnoses are possible. Assessment of situational factors and family characteristics (violence, abuse, neglect) is recommended; ^*4*^Symptoms are alarming with regard to CSA. CSA would be a likely diagnosis, and further assessment to exclude CSA is needed. Assessment of all family and environmental factors and a report to child protective services is recommended.*

### Quantitative Analysis of Parent Reports (Research Question B)

Children aged 7 years or older at time of the OPD evaluation (*n* = 5) were excluded for this analysis, as the criteria in Table 1 did not apply to them. Analyses were performed using SPSS 24.0. Outcome variables were any sexual behaviors reported by parents, and these were assessed according to the categories non-worrisome, somewhat worrisome, worrisome, and very worrisome behaviors (Table 1). Chi-squared tests were used for comparisons between boys and girls. *P*-values of <0.05 were considered statistically significant.

## Results

### Demographic and Abuse-Specific Information (Table 2)

**Table 2:** Demographics.

Sample		*N* total	*n* confirmed CSA	*n* suspected CSA
Number	Total	125	54 (43.2%)	71 (56.8%)
	Males	76 (60.8%)	43 (79.6%)	33 (46.5%)
Median age, min–max^a^		3.3, 0-11^b^	3.0, 0-6	3.0, 0-11^b^
**Family composition^1^**				
Parents married or cohabiting		93 (74.4%)	43 (79.6%)	50 (70.4%)
Parents separated (shared care)		5 (4.0%)	3 (5.5%)	2 (2.8%)
Parents separated (one-parent care)		11 (8.8%)	3 (5.5%)	8 (11.2%)
Blended family		8 (6.4%)	1 (1.8%)	7 (9.8%)
Mean number of siblings (biological)		0.85 (0-2)	0.90 (0-2)	0.82 (0-2)
**Life events^2^**				
None reported		24 (19.2%)	35 (64.8%)	53 (74.6%)
Yes	Any	74 (59.2%)	39 (72.2%)	45 (63.4%)
	Birth of sibling	7 (5.6%)	4 (7.4%)	3 (4.2%)
	Moving house	4 (3.2%)	0	4 (5.6%)
	Pregnancy or fertility problems of parents	8 (6.4%)	5 (9.3%)	3 (4.2%)
	Relational problems of parents	3 (2.4%)	2 (3.7%)	1 (1.4%)
	Physical or psychosocial illness of a parent	12 (9.6%)	6 (11.1%)	6 (8.5%)
	Loss of relative	8 (6.4%)	5 (9.3%)	3 (4.2%)
	Physical illness of child	3 (2.4%)	1 (1.8%)	2 (2.8%)
	Work-related problems of parents	3 (2.4%)	2 (3.7%)	1 (1.4%)
	Other	9 (4%)	7 (12.9%)	2 (2.8%)
	Combination	20 (16%)	9 (16.7%)	11 (15.5%)
**Parental experience of CSA^3^**				
No		32 (25.6%)	23 (42.6%)	9 (12.7%)
Yes	Mother	17 (13.6%)	9 (16.7%)	8 (11.3%)
	Father	1 (0.8%)	1 (1.8%)	0
	Both	1 (0.8%)	0	1 (1.4%)
**CSA-specific information**				
Nature of CSA^c^	Exposure of genitals to child	-	49 (80%)	-
	Ejaculation onto child	-	38 (82%)	-
	Fondling	-	53 (79%)	-
	Oral copulation	-	29 (83%)	-
	Penetration of anus or vagina with finger, penis, or sex toy	-	17 (88%)	-
Frequency of CSA^d^	1-2 times	-	18 (33.3%)	-
	>2 times	-	12 (22.2%)	-
	>5 times	-	11 (20.4%)	-
	>10 times	-	9 (16.7%)	-
	Unclear	-	4 (7.4%)	-
CSA confirmed on basis of perpetrator’s statements only		-	27 (74%)	-
CSA confirmed on basis of perpetrator’s statements and pornographic material		-	27 (85%)	-
Estimated median age at CSA onset, min–max ^d4^		-	1.0, 0-3	-
Estimated median age at CSA cessation, min–max ^d5^		-	2.0, 0-5	-
Mean delay (years) between last abuse and primary assessment, min–max^5^		-	1.2, (0-3)	-

*^*a*^At time of the primary evaluation at outpatient department; ^*b*^One child aged 11 included; ^*c*^Some children victims of multiple types of CSA; ^*d*^Based on perpetrator’s statements; ^*1*^Missing data = 8; ^*2*^Missing data = 27; ^*3*^Missing data = 52; ^*4*^Missing data = 1; ^*5*^Missing data = 6.*

Informed parental consent was obtained for 125 of the 130 children assessed at the emergency OPD, and all of those were included in the study (76 boys, 60.8%, and 49 girls, 39.2%; median age 3.3 years; age range 0–11). Of this sample, 43.2% (*n* = 54) were confirmed victims of CSA (43 boys, 79.6%, and 11 girls, 20.4%; median age 3.2 years; age range 0–6) (Table 2).

#### What Types of Sexual Behaviors and Knowledge Were Reported by Parents of Young Children Assessed for CSA (Table 3)?

**Table 3:** Themes derived from parent reports.

Theme	Category	Specifics	Excerpts from reports
Sexual behavior	Self-stimulation	Easily corrected by parents and observed limited times	“[Girl, 5, suspected CSA] Plays with herself regularly, no clear self-stimulation, no perspiration, no red face.”
		Directed toward objects or other people	“[Boy, 5, suspected CSA] Lying on a teddy bear at the daycare making hip movements resembling a sex act, very hyperactive, trouble concentrating.”
		More extreme, obsessive and/or autoerotic in nature	“[Boy, almost 4, suspected CSA] Often exhibiting masturbation behavior whereby he tended to hump up against everything, at times so forcefully that his foreskin turned red and he couldn’t urinate.”
	Touching other people on private parts	Involvement of siblings or other relatives, playful in nature	“[Girl, 4, suspected CSA] Wanted to play with her little brother’s penis while they were in the bathtub with their mother.”
		More sexual in nature	“[Boy, 5, suspected CSA] During the time when the chief suspect was working in the group, showed transgressive behavior toward older brother in particular, licking and sucking on his arms, belly, breast, and back.”
		More aggressive in nature	“[Boy, 5, suspected CSA] Frequently trying to grab the mother’s ex-partner in the crotch, and aggression toward other children during that period.”
		Involvement of strangers or non-related people, without actual sexual actions	“[Boy, 7, suspected CSA] Touching the breasts of his father’s brother’s wife, whom he hardly knew because she lived abroad.”
	Imitation of sexual acts	More explicit sexual intentions	“[Boy, 5, suspected CSA] Discovered in the shower squatting with his mouth against younger cousin’s penis and giving it a lick.”
	Provocative behavior	Exhibiting own genitals or buttocks	“[Boy, 5, suspected CSA] When naked, proudly shows his penis to mother and her partner.”
		Sexually suggestive posing	“[Boy, 5, suspected CSA] Exhibited enticing behavior in the bathroom, at which time he was in his own world, and he was 4 years old.”
	Defecation-related behavior	Showing off stools	“[Boy, 2½, suspected CSA] Concerns about sexual behavior, gets erections unusually often, sticks his finger in butt crack and shows the feces, wants to be kissed on his mouth lately.”
		Playing with feces	“[Boy, almost 4, confirmed CSA] Obsessed with feces and buttocks and also wants to rub feces onto his penis; in the period when he had contact with the chief suspect, the boy threw dirty diapers around and smeared feces in his bed, and he did that at the daycare center too.”
	Other behaviors	Suddenly acting more prudish than usual	“[Boy, 3, suspected CSA] Abruptly prudish behavior and didn’t want anybody looking at his penis.”
		Going into a trance	“[Boy, almost 4, suspected CSA] When his diapers were changed and his penis was washed, sometimes rolled his eyes and appeared to quite enjoy it.”
Sexuality-related fears and anxiety	Fears or anxieties in child in relation to sexuality or genitals		“[Girl, 2, confirmed CSA] While bathing with her father, remarked ‘I’m scared of the pino’ (the term the family used for the male genital), and that happened another time too.”
			“[Boy, 4, confirmed CSA] Noticeably panics and gets angry if his penis gets hard, as when sitting in a car safety seat.”
Utterances	Sexual slang		“[Boy, 5, suspected CSA] Remarks to daycare teachers: ‘sexy!’ ‘tits!’ ”
	Not clearly sexual utterances		“[Boy, 4, suspected CSA] While mother was changing his baby brother’s diapers, suggested that she lay the baby on the bed naked. Why? Because that’s nice. Why? It’s nice and warm.”
			“[Girl, 4, suspected CSA] Remarks to her mother ‘Pee on your face.’ ”
	More sexually explicit utterances, referring to sexual acts		“[Boy, 4½, confirmed CSA] Said, ‘Daddy should go stick his peepee in Mommy’s mouth.’ ”
		Combined with sexual behavior	“[Boy, 9, suspected CSA] Was reported to have once said to his little brother, ‘You go stick your peepee in sister’s mouth and she’ll slurp on it’; and the brother actually did that, and they had a helluva time.”
	Pain in the anogenital area		“[Girl, 5, confirmed CSA] Says her peepee hurts when she’s urinating.”
			“[Boy, 3½, suspected CSA] Recently indicated pain in his buttocks.”
			“[Girl, 3, suspected CSA] When she was called into the consulting room and was told she’d be examined by the doctor, she spontaneously remarked twice, ‘I won’t let anybody hurt me.’ ”
	Perpetrator-related		“[Girl, 2, suspected CSA] Recognized chief suspect on TV, the picture from the infant daycare, and when asked whether that man was nice, the girl said he was not nice, a naughty man, would start giving her kisses.”
		Might have alluded to sexual activities at the daycare center	“[Boy, 5, confirmed CSA] And a 4-year-old fellow pupil had been told to undress in front of the chief suspect and then hold their penises up against each other’s buttocks. The 4-year-old had told this to his mother, saying that it had happened once and that the chief suspect had said it was good behavior. The point in time when the story was told coincided with the onset of the 5-year-old’s behavioral problems.”

The medical files of 48 children contained parental reports of sexual behaviors or sexual knowledge. Three themes were identified: sexual behavior, sexuality-related fears and anxiety, and utterances reported by parents.

##### Sexual behavior

We identified six categories within the sexual behavior theme: self-stimulating behaviors, touching other people, imitation of oral sexual contact, provocative behaviors, defecation-related behaviors, and other behaviors.

##### Self-stimulating behavior

Self-stimulating behavior was reported by parents during some period of their child’s life. It was manifested in the sample in varying degrees of intensity. In large part, self-stimulating behaviors were easily corrected by parents and were observed limited numbers of times. In some cases, behaviors were directed toward objects or other people. In a few cases, the self-stimulation was more intense and was obsessive and/or autoerotic in nature.

Parents also reported their ***child touching other people***. In the majority of cases this involved siblings or other relatives and was playful in nature. In some cases it was more sexual or aggressive, or it even involved strangers or non-related people. It could consist of touching other people’s private parts either without actual sexual actions or with apparent sexual intentions. ***Imitation of oral sexual contact*** (as described in Table 3) was observed by parents of six children. Another issue relating to sexual behavior was ***provocative behavior***, with some children exhibiting their own genitals or buttocks, and others striking sexually suggestive poses. ***Defecation-related behavior*** was cited for many children [see also (Vrolijk-Bosschaart et al., 2017b)], ranging from resistance to diaper changing to showing off stools or playing with feces. ***Other sexuality-related behaviors*** were mentioned only once or twice, including suddenly acting more prudish than usual or going into a trance.

##### Sexuality-related fears and anxiety

This is a more limited theme, but clearly identifiable nonetheless. Some parents reported fears or anxieties in their child in relation to sexuality or genitals.

##### Utterances

The third theme identified in the parent reports involved “sexual” utterances made by children. A wide range of such utterances were reported by parents during the OPD medical assessment. We distinguished these into four categories: sexual slang, age-inappropriate sexual knowledge, anogenital pain, and perpetrator-related remarks.

##### Sexual slang

Sexual slang included instances of children suddenly using other terms for genitals than those they had learned at home or in their close personal surroundings. Utterances could also reflect ***age-inappropriate sexual knowledge***. Though not all utterances were clearly sexual, remarks referring explicitly to sexual acts were also reported, most often suggesting oral contact. Sometimes these did not remain limited to utterances alone, but culminated into sexual behaviors like those described in Table 3. Many utterances referring to ***pain in the anogenital area*** were reported. Some parents reported children’s utterances that were directly ***related to the perpetrator***, raising concerns in the light of what was later known; other children had made remarks which, in retrospect, might have alluded to sexual activities at the daycare center.

#### In What Cases Were Parent-Reported Sexual Behaviors and Knowledge Worrisome (Table 4)?

**Table 4:** Prevalence of sexual behavior reported by parents.

		Mean age in years, min–max, *SD*	Sexual behavior reported			Age-appropriate behavior			Somewhat worrisome behavior			Worrisome behavior			Very worrisome behavior		
			*n*	%		*n*	%		*n*	%		*n*	%		*n*	%	
Total *N* = 120^1^	Yes	3.6, 1–6, 1.47	48	40		13	27		17	36		16	33		2	4	
	No	2.8, 0–6, 1.45	72	60		–	–		–	–		–	–		–	–	
			*n*	%^2^	%^3^	*n*	%^2^	%^3^	*n*	%^2^	%^3^	*n*	%^2^	%^3^	*n*	%^2^	%^3^
Gender	Girls (*n* = 47)	3.4, 1–6, 1.49	22	46	47	8	17	17	9	19	19	5	10	11	–	–	–
	Boys (*n* = 73)	3.8, 1–6, 1.45	26	54	36	5	10	7	8	17	23	11	23	15	2	4	3
			*n*	%^2^	%^4^	*n*	%^2^	%^4^	*n*	%^2^	%^4^	*n*	%^2^	%^4^	*n*	%^2^	%^4^
Victim status	CSA – suspected (*n* = 66)	3.6, 1–6, 1.56	33	69	50	8	17	12	14	29	21	10	21	15	1	2	2
	CSA – confirmed (*n* = 54)	3.5, 1–5, 1.30	15	31	28	5	10	9	3	6	6	6	13	11	1	2	2

*^*1*^*n* = 5 Excluded from analysis due to age above 7 years at time of abuse assessment; ^*2*^Percentages of total sample for whom sexual behavior was reported (*n* = 48); ^*3*^Percentages of total girls (*n* = 47) and total boys (*n* = 73); ^*4*^Percentages of total children with suspected (*n* = 66) and confirmed CSA (*n* = 54).*

For this analysis we included 120 children (5 children were excluded due to their older age at time of the OPD evaluation, as described under Methods; none of them were confirmed victims). For 40% (*n* = 48) of the children, parents had reported some kind of sexual behavior or knowledge observed in their child (mean age 3.6 years, *SD* = 1.47; 54% (*n* = 26) boys; 31% (*n* = 15) confirmed victims). No significant differences were found between boys and girls (chi-squared test). Sexual behavior or knowledge was considered non-worrisome in 27% (*n* = 13) of the children, somewhat worrisome in 36% (*n* = 17), worrisome in 33% (*n* = 16), and very worrisome in 4% (*n* = 2). Table 4 gives separate overviews of these findings for the confirmed and the suspected victims.

#### How Did Young Children Assessed for CSA Respond Verbally and Non-verbally During Child Interviews (Table 5)?

**Table 5:** Themes derived from child reports.

Theme	Category	Excerpts from reports
Behavioral reactions	Hyperarousal	“[Boy, 4, confirmed CSA] Acts clownish, e.g., shouts a bastardized version of his little brother’s name, climbs up and stands on a chair; when shown pictures of nude children or adults, he averts his eyes and refuses to talk, seeks diversions, ‘Hey look, a car,’ and doesn’t make an impression of being open-minded and engaged.”
	Theatrical/clownish behavior	
	Sexual behavior	“[Girl, 3, suspected CSA] When shown pictures of nude people and people touching each other, she got all giggly and agitated, stood up and began leaning with her crotch against the knee of the woman doing the interview.”
	Specific focus on genitals	“[Boy, 3, suspected CSA] When shown pictures, it was noticeable that he gave primary attention to the genitals and very little to other parts of the body; in response to a picture of children taking a bath, he got somewhat excited, pulled down his pants and diaper, and took hold of his penis.”
	Shutting down	“[Girl, 3, suspected CSA] Showed flighty behavior, was easily distracted and difficult to manage. Abruptly left the room several times to say or show something to her parents. When shown a picture of nude children, she noticeably shut herself off and no longer responded. And when questions were asked about subjects that were potentially emotionally charged, she also didn’t answer the questions and began talking about other subjects.”
		“[Boy, 3, confirmed CSA] Turned quiet and shy when shown a picture of nude children, wouldn’t say much, and would say nothing at all any more after the picture of nude adults.”
Emotional reactions	Anger and aggression	“[Boy, 4½, confirmed CSA] Reacted angrily when shown a picture of nude adults and said, ‘A dick is real real dirty,’ and from then on he didn’t cooperate well and became dejected, sad, and cross.”
	Sadness	
	Fears and anxieties	“[Boy, 6, confirmed CSA] In the stories he told when shown the pictures, issues surfaced such as fear of pain during diaper changing and genitals getting touched against your will.”
		“[Boy, almost 4, confirmed CSA] Told the interviewer he was afraid of the monster, it had a big head, fat belly, and huge buttocks smeared with poop. He found it scary, and he seemed tense while talking about the monster. He called it a poop monster and said the poop comes out of a cave. Asked whether somebody had ever hurt him, he nodded, and when asked where that was, he pointed to his buttocks and murmured ‘heinie.’ When the interviewer asked if he could tell more about that he responded aversively and said we should play and draw pictures and not talk.”
	Inhibitions with regard to sexual acts	“[Boy, almost 4, confirmed CSA] Doesn’t know the difference between boys and girls; asked what you can do with a peepee, his spontaneous reaction is that you’re not allowed to touch it, ‘My little brother starts touching my peepee; that’s a no–no.’ ”
Verbal reactions	Pain	“[Girl, 5, suspected CSA] When shown a nude boy and girl, replies, ‘When you’re at home they can’t hurt you any more,’ and she relates that boys sometimes kick her in the legs or the crotch and she hates that.”
	Unwanted, unpleasant activities	“[Boy, 4, confirmed CSA] Made an uptight, rather over-serious impression. Wouldn’t talk about nudity or intimacy. When shown picture of children playing, he said they’re playing ‘pull the peepee’ and that he didn’t like it because it hurt. Shown a picture of a father putting a child to bed, he said that the father is going to kiss the girl on the mouth, and that the girl doesn’t like that at all. He viewed the pictures fixedly, not looking up at the interviewer. Up until the photos of people in the nude, he had steadily explained what you could do with the various parts of the body shown, but when it came to the penis he said he didn’t know. When asked if he didn’t know or didn’t want to say, he replied that he didn’t want to say. The same applied to pictures depicting any kind of intimacy.”
	Expression age-inappropriate sexual knowledge	“[Boy, almost 5, confirmed CSA] Says that a penis will grow, and that that comes from eating food. You can use it to pee and poop, but he doesn’t approve of touching it (in response to several pictures).”
		“[Boy, 4, confirmed CSA] Speaks normally when shown the first few pictures, but when shown nude pictures he responds that he doesn’t like penises, that they are dirty and have goo on them and can get very big. He decidedly turns away from the pictures, hunches up and looks off in the other direction. The interviewer asks what color the goo is and he replies that it’s white, it’s spit, and he then begins showing distractive behavior. In response to virtually all pictures he says he doesn’t like other people touching a penis. Shown a picture of a child crying in bed, he envisions screaming very loudly and has lost his parents.”
	Don’t want to tell or Don’t know	“[Boy, 6, suspected CSA] Regularly answers during the pictorial interview, ‘Dunno what they’re doing’ or ‘Dunno what that is.’ ”
		“[Boy, 3, confirmed CSA] Basically quite open-minded and engaged, chats away easily, except he won’t answer the question about what a penis is for (doesn’t know at first, and after further probing says he doesn’t want to answer).”

A total of 89 of the 125 children were interviewed by a child behavior specialist in the absence of their parents. The SKPI (Brilleslijper-Kater and Baartman, 2000; Brilleslijper-Kater et al., 2004) was administered to 26 of those children. When we examined the children’s files for our qualitative study, we found salient observations in the reports on 41 children ranging in age from 1½ to 9 years. We distinguished between behavioral, emotional, and verbal sexual reactions. Because reactions in most children were often a combination of these, the frequencies of the three types of observed reactions could not be reliably determined.

##### Behavioral reactions

We distinguished five categories of behavioral reactions: hyperarousal, theatrical/clownish behavior, sexual behavior, specific focus on genitals, and shutting down. Most children showed more than one category at once, as illustrated by the file excerpts in Table 5.

Some children tried to distract the interviewer and showed signs of both hyperarousal and theatrical/clownish behavior, or both hyperarousal and sexual behavioral expression, in response to the interview methods.

Some children appeared to have a specific focus on genitals, sometimes in combination with sexual behavior. Other children showed rather aversive behavior, such as shutting down. Abrupt behavioral changes from open-minded to withdrawn were also observed.

##### Emotional reactions

Emotional reactions discerned in children were anger, aggression, sadness, fears, and anxieties. In some children, emotions appeared to be triggered; for others they seemed part of a more general aura. The first example in Table 5 shows both anger and aggression in combination with sadness, seemingly triggered by nudity.

This contrasts with children whose overall aura was more emotional and for whom emotions were a recurrent theme in their reactions and stories. Some children talked clearly about their fears and anxieties. Some expressed inhibitions with regard to sexual acts.

##### Verbal sexual reactions

The third salient theme identified from the child interviews involved verbal “sexual” reactions by children. These consisted either of utterances with actual information or of explicit refusals to tell. Utterances particularly noted by the interviewers were about pain, unpleasant and unwanted actions, and sexual knowledge.

Pain was a subject mentioned several times by interviewed children. Sometimes it involved children hurting each other. Some children referred to unwanted and unpleasant actions by adults. Some also showed a reluctance or refusal to talk about certain issues.

The interviewers noted a number of children who expressed sexually inappropriate knowledge (in view of their age and development). Some examples were ambiguous, and others much clearer.

Many children explicitly did not want to talk. The child behavior specialist noted that some children would start the interview with an open-minded and engaged attitude and would suddenly become restrained and uncommunicative when nudity, genitals, or other sexually related topics were broached. They used phrases like “I don’t want to tell…” or “I don’t know.”

Some children showed distinctive behavioral, emotional, and verbal sexual reactions in combination, as in the following file excerpt: “[boy, 5, suspected CSA] puts his hands over his eyes and turns his body away when shown a picture of two nude adults. He says he does not want to see them because they are disgusting and filthy. At the same time, he gives striking interpretations of other pictures: on seeing a picture of a man with a child, he mentions a baby-changing pad; and after a picture of a woman helping a child to urinate, he says that adults make children feel scared and hurt them and that children don’t like it. He remains sitting with his hands before his eyes and his body turned aside until the interviewer puts the pictures away and changes the subject.”

## Discussion

In this mixed-methods study, we analyzed the kinds of sexual behavior and knowledge reported by parents of young children in assessments made after incidents of suspected or confirmed CSA. We then sought to determine how many such cases were worrisome according to pre-established criteria. We further investigated how young children assessed for CSA responded verbally and non-verbally during child interviews.

In qualitative analyses of the parent reports, we identified three themes: (1) sexual behavior (self-stimulation, touching other people, imitation of oral sexual contact, provocative behaviors, defecation-related behaviors, and other behaviors); (2) sexuality-related fears and anxiety (a more limited, but clearly identifiable, theme); and (3) sexual utterances (sexual slang, manifestations of sexual knowledge, and references to sexual acts, anogenital pain, or the perpetrator). In the child interviews, we also identified three themes – behavioral, emotional, and verbal reactions – sometimes also observable in combination.

Of the 48 children for whom sexual behavior or knowledge was recorded in the parent reports, it was judged by the experts to be worrisome or very worrisome in 37% of the cases (*n* = 7 confirmed victims and *n* = 11 suspected victims). In a comprehensive review in 1993, which also included young children (<6 years of age), sexual behavior problems were reported in 28% of the CSA victims (Kendall-Tackett et al., 1993).

Although sexual behavior problems are reported more often in CSA victims than in non-abused children, there is no single specific behavior that is indicative of sexual abuse (Kellogg et al., 2009). Other origins of developmentally inappropriate sexual behaviors, including physical abuse, family violence, and other types of maltreatment, are known to be possible (Brilleslijper-Kater et al., 2004; Chaffin et al., 2008; Kellogg et al., 2009; Everson and Faller, 2012; Allen, 2017). All the same, evaluation of a child’s sexual behavior is recommended as part of the assessment for CSA, and it is therefore important that pediatricians who perform such evaluations be well aware of normal, developmentally appropriate variations in children’s sexual behavior (Jenny and Crawford-Jakubiak, 2013).

As a general principle, sexual behavior needs to be evaluated in the full context of each presenting case. The interpretation of sexual behavior is dependent on contextual factors such as the child’s age and social, cultural, and family background (including modes of childrearing and approaches to sexuality), as well as the rapidity of onset (perhaps reflected in sudden changes in behavior). Friedrich and colleagues also established a range of other factors that may influence children’s sexual behavior: situational factors (e.g., child developmental level, environmental changes), maternal education, family sexuality, family stress, family dysfunction, family violence, and hours per week in daycare (Friedrich et al., 1998). Whether or not sexual behavior and knowledge should be deemed worrisome also depends on whether such behavior and knowledge is reported in combination with other psychosocial symptoms (Vrolijk-Bosschaart et al., 2017b).

Age is an essential factor to consider when evaluating sexual behavior in young children. Younger children may have difficulties expressing what has happened to them, because the events do not fit into their reference frame. One single sign or symptom may raise serious concerns in a 1-year-old, whereas the same issue by itself would not be alarming in an older child. Age-inappropriate sexual behavior in children (both non-abused and abused) is probably related to life stressors, including CSA. The older a child, the more problems that can come to light, by virtue of older children’s abilities to express themselves and also the broader options for examination. In very young children, one must rely more on behavioral observations (Brilleslijper-Kater et al., 2004). However, as Friedrich and colleagues reported, sexual behavior shows an inverse relationship with age, dropping off after the age of five (Friedrich et al., 1998). The frequency in which sexual behavior is exhibited thus diminishes as children get older.

### Sexual Knowledge in Children

Worrisome sexual knowledge in our sample was often manifested in children’s utterances, although the actual meaning of some utterances was unclear or debatable. In the unique case we studied, we possessed information on the context of the abuse. This was helpful to experts in clarifying the meanings of children’s utterances. Some children made explicit sexual utterances referring to sexual acts, most often oral ones. Sometimes that was not limited to utterances alone but culminated in actual sexual behaviors. Utterances referring to actual sexual acts normally do not belong to young children’s frames of reference with respect to sexual knowledge (Brilleslijper-Kater and Baartman, 2000; Brilleslijper-Kater et al., 2004).

On the whole, young children have very little sexual knowledge. They only possess certain basic knowledge of genital differences, gender, sexual body parts, and non-sexual functions of the genitals. Knowledge of pregnancy, birth, reproduction, and adult sexual behavior is very limited and decreases in that order. Younger children generally know less than older ones (Brilleslijper-Kater and Baartman, 2000).

There are several reasons why sexual knowledge may be a more useful discriminant for assessing CSA than sexual behavior. Sexual knowledge can be more objectively quantified, and measurement need not depend on caregivers’ observations. And although the extent of knowledge may derive partially from the extent of experience, knowledge of sexuality is likely to be less dependent on abuse-related variables (Brilleslijper-Kater et al., 2004). There could be a multitude of reasons why sexual knowledge differs between children, such as differences in parentally provided sex education, exposure to sexuality-related information from peers and siblings, and exposure to sexual media. Such qualifications would limit the conclusions that could be drawn from an assessment indicating advanced sexual knowledge.

To be able to express sexual knowledge, children need a certain level of speech development. Clinicians must keep in mind that, even if young children are talking, they still may not have the words to express their experiences. For example, young children do not know words like ejaculation, so they may describe ejaculation as “peeing,” a word they do know (Brilleslijper-Kater et al., 2004).

Unfortunately, children’s sexual knowledge has been studied much less extensively than sexual behavior (Brilleslijper-Kater and Baartman, 2000; Brilleslijper-Kater et al., 2004). More research is needed on age-appropriate sexual knowledge, on differences in sexual knowledge between abused and non-abused children, and on how to quantify children’s sexual knowledge.

### Child Interviews

As one component of our observations of some children from our sample, we used the SKPI as the basis for a semi-structured interview. Previous research has shown that non-abused children respond with an open-minded and engaged attitude to the interview material and that sexually abused children show significantly more non-verbal reactions indicative of avoidance of the sexual meanings of pictures (Brilleslijper-Kater and Baartman, 2000; Brilleslijper-Kater, 2005). The SKPI seems a helpful tool to test children’s sexual knowledge and to assess them for non-verbal signs of CSA. Unfortunately, the SKPI has thus far not been validated as a tool to differentiate between abused and non-abused children. To our knowledge, no other such interview tool exists as of yet. Research to validate the SKPI is now in progress.

The administration of non-verbal techniques like the SKPI in abuse interviews has been widely debated. The use of anatomical dolls, for example, is thought to encourage leading or suggestive lines of questioning, as well as overinterpretation of a child’s play (Everson and Boat, 1994). However, when non-verbal techniques are employed in accordance with a standardized protocol, using open and non-suggestive questions, they can be valuable instruments, especially in young children with limited verbal abilities (Everson and Boat, 1994). Professionals need to be trained to use such instruments adequately and use forensic interview techniques to minimize influencing and leading the children who are being interviewed.

Another important point of discussion is the possibility that, by using the SKPI, we could have induced secondary trauma in CSA victims. As the SKPI contains solely child-friendly drawings, we believe the risks of secondary traumatization were very limited.

For the above named reasons we chose to only qualitatively analyze the children’s responses during the interviews. More research is needed on the reliability and validity of the use of the SKPI in assessing alleged CSA in children. Until then we need to be careful in interpreting our findings.

### Strengths and Limitations

This study presents unique data from a sample of 125 young children who were assessed for suspected CSA. For the 54 confirmed victims, a high level of evidence was available (police reports including perpetrator’s statements and detected pornographic images). The sample originates from a naturalistic setting and thus reflects the dilemmas in daily practice. Our sample consisted mostly of very young children, predominantly boys. Research on signs and symptoms of sexual abuse in this specific group is valuable because knowledge in that area is scarce. The study methods yielded an overview of the varied sexual behavior in young children assessed for CSA.

We should nonetheless acknowledge some considerable limitations. First, potential bias existed on the part of the responding parents, the clinicians who performed the initial assessments in 2010, and the researchers. All parents knew that their child was being assessed for possible CSA, and parents in our study may hence have reported more problems than they normally would have. Parents may have either overestimated or underestimated problems in their child when interviewed. They may have highlighted behaviors that would otherwise be considered normal, or, as a result of the commotion, they may have not been complete in reporting signs and symptoms.

Secondly, although the clinicians involved in the evaluations were all experienced in evaluating suspected CSA, none had ever experienced a sexual abuse case on this scale. As time passed, more and more information about the perpetrator and his actions became publicly known. This could have introduced bias to those clinicians assessing the children.

Thirdly, during the qualitative and quantitative analysis the researchers knew that the data they were analyzing originated from the ASAC. We tried to minimize bias by having two independent researchers to review the data.

We used the children’s medical files for data extraction. It should be noted that the clinicians who performed the original examinations inevitably made selections of data from the full assessment to include in their file notes, deciding which findings and utterances they believed relevant to record. The medical files were built on retrospective reporting, which could have introduced recall bias. Often it was difficult to assess how frequently a child had exhibited certain behaviors and to assign the behaviors to the four categories (see Table 1). Additionally, we were not able to assess the sexual knowledge the children might have acquired at home, nor how long the parents observed their children’s non-appropriate sexual knowledge and behaviors and how they responded to it, because this was not reported in the medical files.

We chose to limit ourselves to a qualitative description of the child interviews. Evidence to establish criteria for quantitative analysis is still too limited, and the SKPI used in some of the interviews is not yet a validated tool. One should therefore be cautious in interpreting our findings. Due to the urgency with which the OPD was set up, only written reports of the child behavioral specialists were made. Had the child interviews been video-recorded, more quantitative data could have been obtained.

This acute setting at the time of the initial assessment resulted in missing data on some important potential confounders, such as histories of other types of maltreatment, other life events, and parental experiences of CSA; we were therefore not able to control for such issues.

Ideally, we would have compared our findings to a sample of non-abused children who were not involved in the ASAC, but we could not obtain approval from the Medical Ethics Committee to include a control sample. Comparing confirmed victims to suspected victims was not feasible for two reasons: the group with suspected CSA likely contained children who were actual victims, and children who were not abused themselves may have witnessed CSA in other children, possibly their siblings. The perpetrator’s statements indicated that the abuse took place at the daycare center or in children’s homes while babysitting, and it is therefore likely that children witnessed other children being sexually abused.

Further, we need to address that there are some limitations to the generalizability of the outcomes of this study. First, the systematic abuse at the daycare center (and at home) means that the children involved (and their parents) lived among other children who have been abused. Second as all abuse was perpetrated by one particular abuser, and children’s sequelae might be related to the abuser’s modus operandi. Thirdly, this sample does not represent the ethnic variation in other clinical settings as all children lived in the same area. This study of a large sample of confirmed or highly suspected CSA victims is unique. One should nonetheless be cautious in applying the outcomes to the general population, because our sample represents a non-randomly selected group of children from Amsterdam and neighboring communities in the Netherlands.

### Future Perspectives

To our knowledge, there is not yet any generally accepted, scientifically valid way to diagnose or exclude CSA in young children (Kendall-Tackett et al., 1993; Finkelhor and Berliner, 1995; Duffy et al., 2006).

Most research appears to focus on children’s sexual behavior rather than their sexual knowledge (Brilleslijper-Kater and Baartman, 2000; Brilleslijper-Kater et al., 2004). We would argue that children’s sexual knowledge could be a more objective and more readily quantifiable indicator than their sexual behavior (Brilleslijper-Kater et al., 2004). More evidence is needed about age-appropriate and age-inappropriate sexual knowledge, as well as more research on how to assess sexual knowledge, especially in preschool children. Assessment could be difficult, as there are various reasons why sexual knowledge differs between children which may be unrelated to abuse, such as differences in sex education, differential exposure to sexuality-related information from peers and siblings and differential exposure to sexual media. Such difficulties would limit the conclusions that could be drawn from any indications of advanced sexual knowledge. Despite those limitations, future research should seek instruments to measure children’s sexual knowledge. Non-verbal techniques such as the SKPI must be evaluated further with the aim of creating standardized, validated interview techniques for young children under examination for possible CSA.

We would recommend that future research focus on both sexual behavior and sexual knowledge, and on using parental reports and child interviews in conjunction. A better understanding of age-appropriate and age-inappropriate sexual behavior and knowledge in young children will enable us to improve the assessment of suspected CSA.

Most often, researchers use preselected CSA victims and preselected controls in their studies (Vrolijk-Bosschaart et al., 2018). A different methodology would be required to study whether presenting symptoms can be accurately differentiated between those of sexually abused and those of non-abused children. Thus, there is a need for research using a sample of children with suspected CSA. The greatest limitation remains that there is no real “gold standard” to determine or rule out CSA, unless police evidence such as perpetrator confessions, DNA traces or pornographic images is available, and that is rarely the case in CSA.

Diagnosing CSA in young children is difficult. Systematic clinical, preferably multidisciplinary, evaluation needs to be performed. Wallace and colleagues have shown that a multidisciplinary approach for assessing child abuse is effective (Wallace et al., 2007). The American Academy of Pediatrics recommends that pediatricians “seek a second expert opinion in cases of CSA when the child’s anal or genital examination is thought to be abnormal” (Jenny and Crawford-Jakubiak, 2013). We would prefer to extend this advice to include multidisciplinary consultations in all cases of known or suspected CSA.

## Conclusion

This unique study – based on a large sample of predominantly male infant and preschool children susceptible to sexual abuse by one convicted perpetrator, including 54 cases of confirmed and 71 cases of suspected CSA – revealed a wide range of sexual behaviors, sexuality-related emotional reactions, and sexual utterances in the children involved. In 37% of the children, those manifestations were considered worrisome. When children present with suspected CSA, we advise clinicians to assess them in terms of sexual behavior problems and inappropriate sexual knowledge, but to keep in mind that there are other possible causes for worrisome sexual behavior and knowledge in children.

## Author Contributions

TV-B contributed to the conception, design, data collection and qualitative and quantitative data analysis, assisted in the design, and wrote the first draft of the manuscript. MdL and MR assisted in the data collection. GW and YV contributed to the conception and design and assisted in the methodological aspects of this study. EV, EvD, AT, SB-K, AV, MB, and RL contributed to the conception and design, and were involved in critically revising the manuscript.

## Conflict of Interest Statement

The authors declare that the research was conducted in the absence of any commercial or financial relationships that could be construed as a potential conflict of interest.

## Abbreviations

AMC: Academic Medical Center, Amsterdam
ASAC: Amsterdam sexual abuse case
CSA: child sexual abuse
FGD: focus group discussion
OPD: Emma Children’s Hospital, Amsterdam, Outpatient Department
